# Low-fat frankfurters from protein concentrates of tilapia viscera and mechanically separated tilapia meat

**DOI:** 10.1002/fsn3.42

**Published:** 2013-10-18

**Authors:** Angela D Cavenaghi-Altemio, Lígia B Alcade, Gustavo G Fonseca

**Affiliations:** Laboratory of Bioengineering, Faculty of Engineering, Federal University of Grande DouradosDourados, Mato Grosso do Sul, Brazil

**Keywords:** Frankfurter, mechanically separated meat, Nile tilapia, surimi

## Abstract

In order to develop a healthy low-fat frankfurter-type sausage, different formulations were developed with tilapia viscera surimi (T1) and two with mechanically separated tilapia meat (MSTM) surimi (T2 and T3), all without pig lard addition. Due to technological problems observed for T1 sausage during cooking, it was not further investigated. The functionality of the other two formulations was evaluated based on proximate composition, pH, water activity, and texture. Finally, microbiological and sensory analyses based on acceptance tests were performed. *Listeria monocytogenes* and *Salmonella* spp. were found to be absent. T2 showed higher frequencies for the attributes color (90.0%) and overall acceptability (86.7%), while T3 showed higher frequencies for taste (86.7%) and texture (96.7%). The surimi concentration was reflected in the physical properties of the sausages. It was found that the addition of MSTM surimi to sausage favored greater cutting strength (3.9 N for T2 and 4.9 N for T3). Beyond the surimi utilization, the total replacement of pig lard by cassava starch and soybean protein had also contributed with the texture properties.

## Practical Applications

High-fat diets are a risk factor for obesity and cardiovascular diseases. Thus, it is important to develop low-fat or reduced-fat food products. The trend is challenging for comminuted meat products because reducing fat often results in changes in palatability of the meat products. The protein quality of several foods is significantly increased when pig lard is substituted by fish protein concentrate into formulations. The benefits are increased if waste protein from the fish processing industry is utilized.

## Introduction

In 2010, Brazil produced about 1.26 million tons of fish, and 38% from aquaculture. Tilapia is the leading aquaculture fish produced in Brazil, with more than 132 thousand tons produced per year (MPA 2012).

Nile tilapia, *Oreochromis niloticus* (L.), is a tropical fish with great potential for aquaculture. It presents rusticity, good growth rate, and adaptability to confinement, producing a tasty white color meat (Oliveira Filho et al. 2010). The industrial processing of Nile tilapia has frozen fillets as a priority. However, the yield on fillet is low (30–35%), resulting in a large amount of waste (Garduño-Lugo et al. 2003; Kubitza and Campos 2006; Oliveira et al. 2006) that may turn into environmental and economic problems.

This solid waste has approximately the same protein content as fish flesh. In order to recover these proteins of high nutritive value for human consumption, different approaches have been described in the literature (Batista 1999). Fish processing wastes including viscera have been reported to be a good source of proteins. Fish viscera constitute approximately 20% of the fresh water fish biomass, and are a rich source of protein and polyunsaturated lipids (Bhaskar et al. 2008).

Among the alternatives for these fish residues is the surimi, which is a semiprocessed, wet, frozen, washed fish myofibrillar protein concentrate (Lanier 1986). It is an odorless and tasteless material (Belibagli et al. 2003) with unique textural properties and high nutritional value (Park and Morrissey 2000). Surimi and surimi-based products have been gaining popularity in recent years for their protein quality, low fat content, long shelf life, and convenience in consumption (Chen 2002; Khan et al. 2003). Tilapia surimi production has been reported elsewhere (Marchi 1997). Another alternative for this waste is the obtaining of mechanically separated fish meat (Finne et al. 1980; Gryschek et al. 2003), with which a variety of products, including frankfurter-type sausages (Oliveira Filho et al. 2010), can be produced.

Consumer preference for healthier products has been promoting the research and development of new foods with less or no meat. Particularly, consumer interest for meat analogs and, also, fat substitutes, has increased (Cardoso et al. 2008a). Concerning fat, it was found that low-fat frankfurters containing soy protein or starch had sensory and texture properties similar to the high-fat products (Yang et al. 2001). This trend is especially challenging for comminuted meat products because reducing fat often results in changes in palatability of the meat products (Wang and Xiong 1999). In this context, the development of restructured fish products and the application of new food ingredients have been used as a way of creating healthier meat substitutes and also as a means to upgrade low-value species and the waste generated by the fish processing industry (Cardoso et al. 2008b).

The aim of this work was to develop frankfurter-type sausages from tilapia viscera (TV) surimi and mechanically separated tilapia meat (MSTM) surimi, and to evaluate its physical, chemical, microbiological, and sensory characteristics.

## Materials and Methods

### TV and MSTM

Fresh TV and MSTM were supplied by a local fish processing industry. They were transported under refrigerated conditions to the laboratory and kept at −18°C before use. The MSTM was produced from fish carcass in 3 mm particle size using a Baader separator (Baader model 694, Lübeck, Germany), operating at an inlet temperature of 6°C and an outlet temperature of 10°C.

### TV surimi and MSTM surimi

TV and MSTM were washed in four and three cycles, respectively, utilizing in each cycle a washing solution:meat (TV or MSTM) ratio of 4:1 (w/v), temperature of 10°C, for 5 min. The stirring was kept constant at 500 rpm. For the initial washings (first, second, and third for TV, and first and second for MSTM) 0.25% NaHCO_3_ solution and for the final washing (fourth for TV, and third for MSTM) 0.3% NaCl solution were used. The centrifugations were carried out at 3500 *g* for 15 min. The supernatant containing fat and water-soluble proteins was discarded. The final slurry was sieved through a 1-mm mesh metal screen to remove connective tissues, packaged in five-layer nylon propylene bags, and stored at −18°C.

### Frankfurters

Frankfurters were prepared using three different formulations (Table 1). Ingredients were supplied by IBRAC Indústria de Aditivos e Condimentos Ltda, Rio Claro, SP. Natural ovine casings (caliber 24-26) were provided by Cavenaghi Comércio de Embalagens e Condimentos Ltda, and cellulose casings (caliber 22-23) by Seara Alimentos, both located in Dourados, MS. Frozen surimi was comminuted in a cutter (Filizola, São Paulo, Brazil) and then transferred to a multiprocessor (Walita, São Paulo, Brazil) where the other ingredients were added. The ingredients were mixed with the surimi prior to preparation of the inlaid emulsion. Cold water was added during the mixture in one of the formulations (T2). The emulsion was stuffed into inlaids, natural ovine casings type, and boiled at 65°C in a water bath until color developed. Thereafter, the cooking temperature was increased to 80°C until the nternal temperature had reached 74°C. The sausages were then water cooled with 5% of the vegetal colorant urucum for 20 min. The casings were dived in 2% phosphoric acid solution (pH 2–3) at 25°C for 4 h. The finished frankfurters were vacuum packed in polyethylene bags, labeled, and stored overnight (12 h) at 2°C for microbial and texture kinetics analysis. For proximate composition and sensory evaluation, frankfurters were stored at −18°C till further analyses.

**Table 1:** Composition of three emulsified inlaid frankfurter-type sausages formulations

Formulation (%)/treatment	T1	T2	T3
TV surimi	91.25	–	–
MSTM surimi	–	76.25	91.25
Cold water	–	15.00	–
Soybean-concentrated protein	4.00	4.00	4.00
Cassava starch	2.00	2.00	2.00
Cure salt	0.25	0.25	0.25
Global sausage SV 800	0.80	0.80	0.80
Cochonilha carmim	0.05	0.05	0.05
Refined salt	1.65	1.65	1.65

T, treatment; TV, tilapia viscera; MSTM, mechanically separated tilapia meat; T1, treatment 1; T2, treatment 2; T3, treatment 3.

### Proximate composition

Moisture, crude protein, and crude fat contents were determined according to the method described by AOAC (1995). Moisture was determined by the oven drying method at 110°C for 24 h; for cooked samples, total water content was calculated as (100 − [total protein + total lipid + total ash]). Total protein content was determined by the micro-Kjeldhal method. Total lipids were evaluated by the Soxhlet method.

### pH and water activity

pH was measured using a digital pH meter (Model PH; Instrutherm, São Paulo, Brazil). The electrode was inserted in six different points of each sample (TV, MSTM, TV surimi, MSTM surimi, or frankfurter), in duplicate, and the pH was recorded. Water activity was measured at 25°C using an electronic device (Model AquaLab; Decagon Devices Inc., Pullman, WA), for each sample (TV, MSTM, TV surimi, MSTM surimi, or frankfurter) in triplicate.

### Texture analysis

Texture analysis of the frankfurter was carried out using a texture analyzer Model TA-HDI (Stable Micro Systems, Surrey, UK). Frankfurters kept at 2°C were equilibrated at room temperature (28–30°C) before analysis. Cylindrical samples, 2.5 × 3.0 cm, were prepared, placed in the texture analyzer, and submitted to a cutting/shearing test using a Warner-Bratzler shear blade (1 mm thick) to determine the cutting strength (N), which indicated the firmness of the sample.

### Microbiological analysis

To assess microbiological analysis, duplicate 25 g samples were aseptically transferred into a stomacher bag containing 100 mL of sterile distilled water containing 0.1% peptone (1% for *Salmonella* spp. determination). Samples were homogenized for 1 min. Ten-fold serial dilution were prepared using sterile 0.1 peptone solution (9 mL), and spread plated (0.1 mL) in duplicate onto broths and/or agars for detection of typical colonies, biochemical confirmation and identification, and plate counting (*Listeria monocytogenes*, *Salmonella* spp, and *Staphylococcus aureus*) (USDA/FSIS 1998).

### Sensory evaluation

Sensory analyses were conducted by 30 nontrained panelists. A 7-point hedonic scale (1 = like extremely; 7 = dislike extremely) was used for evaluation of the overall acceptability. Samples (2-cm-long pieces) were prepared by steeping the emulsified inlaid in boiling water for 3 min, draining the liquid, and holding on a warming tray in covered plates for no longer than 30 min. Panelists were questioned about color, taste, texture, and overall evaluation. The statistical analysis was performed by analyses of variance (ANOVA) using the Statistica v.8.0 software (StatSoft, Inc., Tulsa, OK), and means were compared by the Tukey test (5% probability) using Microsoft Excel. Purchase intention was expressed as the percentage of total score.

## Results and Discussion

### Yield, pH, water activity, and proximal composition

The average yield of MSTM surimi was 31.4 ± 2.3%. This value is slightly superior to the data reported in the literature, which vary between 20% and 25% (Neiva 2010). Average pH and water activity are presented in Table 2. The values for TV surimi were 6.7 ± 0.1 and 0.989 ± 0.008 for pH and water activity, respectively. In most of the cases, higher pH values are obtained for surimi due to the successive washings with sodium bicarbonate.

**Table 2:** Proximal composition, pH, water activity, and cutting strength of mechanically separated tilapia meat surimi and two emulsified inlaid frankfurter-type sausages formulations

Determination	MSTM surimi	T2	T3
Moisture	82.84 ± 0.04	77.76 ± 0.48	73.91 ± 0.35
Protein	11.23 ± 0.09	9.90 ± 1.75	13.29 ± 0.44
Lipids	2.53 ± 0.18	2.56 ± 0.11	2.73 ± 0.19
Ash	1.04 ± 0.13	7.56 ± 0.21	8.00 ± 0.20
pH	7.10 ± 0.04	6.81 ± 0.01	6.71 ± 0.05
Water activity	0.998 ± 0.003	0.987 ± 0.001	0.984 ± 0.005
Cutting strength (N)	–	3.9^a^ ± 0.3	4.9^b^ ± 0.1

Different letters in the same line means that there exist significant difference (*P* > 0.05) by the Tukey test. Without letters in the same line there is no significant difference (*P* > 0.05) by the Tukey test. MSTM, mechanically separated tilapia meat; T2, treatment 2; T3, treatment 3.

Proximate compositions obtained for MSTM surimi are presented in Table 2. These values are very close to that reported for MSTM surimi published elsewhere (Alcade et al. 2010), and several types of sausages (TBCA-USP 2005) and formulations (Andrès et al. 2006).

**Figure 1:**
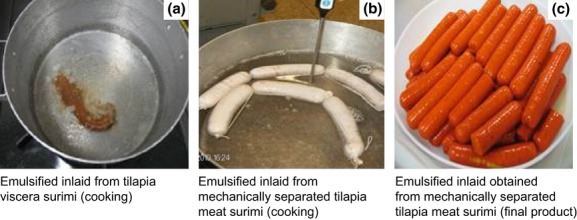
Emulsified inlaid from tilapia viscera surimi (a) and mechanically separated tilapia meat surimi (b) during cooking. Emulsified inlaid (final product) obtained from mechanically separated tilapia meat surimi (c).

The surimi frankfurters presented low lipid content compared with sausages obtained from other meat sources (TBCA-USP 2005), mainly due to the no pig lard addition, and low lipid content of the surimi (Table 1). The moisture and lipid contents of sausages are influenced by the characteristics of the meat utilized for the mechanically separated meat obtaining (Mielnik et al. 2002) and hydroscopic characteristics of the additives (Figueiredo et al. 2002). The high moisture was due to the utilization of surimi as raw meat material, which presented a high capacity of water retention, which is in agreement with work published elsewhere (Cortez-Vega et al. 2013). The reduced moisture for T2 and T3 was due to the addition of additives. For T3 the moisture was even lower due the no addition of cold water during the mixture of the formulation constituents. Ash content was much higher than surimi due to mainly the addition of refined and cure salts. T1 was not further investigated because presented technological problems during cooking (Fig. 1a). Considering that T1 presented the same composition as T3, except the surimi source (TV was utilized for T1 and MSTM for T3), and that surimi was the main constituent of the frankfurter's formulations (Table 1), it can be expected that the high texture was due to the high moisture content, as a consequence of the high content of myofibrillar protein from both raw materials. However, the myosin ratio in surimi products is also dependent on the muscle fiber type in raw muscles (Kang et al. 2010). These differences in texture can be eventually attributed to the structural difference among the heavy chains that compose the myosins and the interaction between myosin and actin (Cortez-Vega et al. 2013).

### Texture analysis

Cutting strength was measured for two different formulations (T2 and T3) of frankfurter-type sausages (Table 2). The cutting strength was 3.9 and 4.9 N for T2 and T3 formulations, respectively, both developed with MSTM surimi. These values are similar to frankfurters obtained from mechanically separated hybrid sorubim meat (4.24 N) and slightly superior to frankfurters obtained from mechanically separated hybrid sorubim meat surimi (2.47 N) (Azambuja et al. 2010).

The protein content in cooked frankfurters was not significantly correlated with rupture force, which suggests that the increased gel rupture force is most likely due to the functional performance of the protein type rather than the protein content (Wang and Xiong 1999). However, here both the increase in gel force (91.25%) and in protein content (13.29%), due to the higher surimi concentration, reflected in a higher firmness of the frankfurter (4.9 N) in T3. In this way, it seems that the utilization of the higher quality protein from surimi increases the desirable functional properties of the frankfurter due to the increased association of water molecules with the proteins of surimi (Stangierski et al. 2008).

Finally it is important to underline that the total replacement of pig lard by cassava starch and soybean protein had also contributed to the texture properties. Low-fat sausage is less hard than high-fat sausage (Carballo et al. 1996) and when the fat level is reduced, there is a significant decline in texture properties of the products. The incorporation of surimi produces scarcely any alteration in the fat and water-binding properties and the rheological characteristics of meat products (Cavestany et al. 1994). However, starch has been recognized as a filler in surimi and used to increase the hardness and firmness of products and enhance the gel strength. It has also been used to improve the texture of low-fat frankfurters, favoring the formation of a more compact and stronger heat-induced protein network (Carballo et al. 1995; Chuapoehuk et al. 2001; Li and Yeh 2003).

### Microbiological analysis

Muscle foods require good pasteurization practices to maintain microbial safety, but it must not compromise the overall quality of the product. There is a distinct difference in heating times for texture development and for microbial safety (Jaczynski and Park 2003) and it must be taken into account during product development.

Pasteurization needs to eliminate targeted bacterial pathogens and reduce spoilage bacteria in order to extend the shelf life of the product. The FDA established a “zero tolerance” for *L. monocytogenes* and *Salmonella* spp. in ready-to-eat products. According to the FDA's informal guidance, the product may be recalled if 10^4^ CFU/g of *S. aureus* are present (Ward and Price 1992). Our microbiological results are in accordance with these exigencies (Table 3) and also with Brazilian legislation, which establishes for *S. aureus* a maximum of 3 × 10^3^ CFU/g, beyond the absence of *L. monocytogenes* and *Salmonella* spp. (Ministry of Health, Brazil 2001).

**Table 3:** Microbiological analysis for two emulsified inlaid frankfurter-type sausages formulations

Microbiological analysis (CFU/g)	T2	T3
*Salmonella* spp	Absence	Absence
*Listeria monocytogenes*	Absence	Absence
*Staphylococcus aureus*	30	50

T2, treatment 2; T3, treatment 3.

### Sensory analysis

In terms of “overall acceptability,” sensory evaluation scores showed that both frankfurters T2 and T3 obtained the highest acceptability scores (50.0%). Table 4 shows the sensorial attribute scores for the acceptation test for the consumers of emulsified inlaid frankfurter-type sausages obtained from MSTM surimi in two different formulations (T2 and T3). It can be observed that there was no significant difference for the attribute scores evaluated at the 5% significance level.

**Table 4:** Sensorial attributes scores for the acceptation test for the consumers of two emulsified inlaid frankfurter-type sausages formulations

Sensorial attributes	T1	T2	Comments
Color	5.9 ± 1.14	5.8 ± 1.30	No comments
Taste	5.2 ± 1.33	5.7 ± 1.18	Reduce pepper; increase salt
Texture	6.0 ± 1.10	6.4 ± 0.94	Refine; increase firmness
Overall acceptation	5.4 ± 1.27	5.7 ± 1.27	No comments

Without letters in the same line there is no significant difference (*P* > 0.05) by the Tukey test. T2, treatment 2; T3, treatment 3.

**Figure 2:**
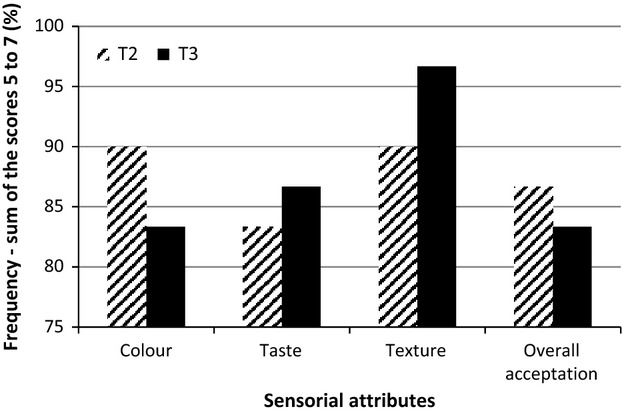
Histogram of the sum of the frequencies (%) of the scores representing acceptation 5 (like slightly) to 7 (like much) for the sensorial attributes “color,” “taste,” “texture,” and “overall acceptation.” T2, treatment 2; T3, treatment 3.

Figure 2 presents the histogram of the sum of the frequencies (%) of the scores representing acceptation from 5 (like slightly) to 7 (like much) for the sensorial attributes evaluated for both treatments (T2 and T3). T2 showed higher frequencies for the attributes color (90.0%) and overall acceptability (86.7%), while T3 showed higher frequencies for taste (86.7%) and texture (96.7%).

**Figure 3:**
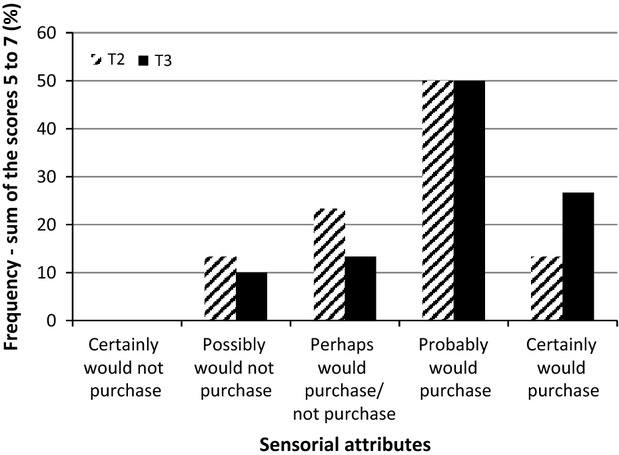
Percentage of purchase intention of the obtained frankfurter-type sausages. T2, treatment 2; T3, treatment 3.

Figure 3 presents the histogram with the percentage of purchase intention of the obtained frankfurter-type sausages from T2 to T3. It can be observed that both formulations obtained the same percentage of purchase intention (50.0%) for probably would purchase attribute. However the intention of certainly would purchase, showed the higher value (26.7%) for T3. Considering the sum of the percentages of the purchase intention attributes of probably and certainly would purchase, T3 showed itself more accepted by the consumers.

Comparing the results obtained from Figures 2 and 3, it is possible to affirm that texture was the sensorial attribute that most affected the preference of the consumers. The addition of MSTM surimi into the formulation showed a good sensory acceptability mainly due to the quality and texture parameters of this surimi. Surimi-like material had been incorporated into restructured products enhancing fat-binding properties in frankfurter without adversely affecting acceptability and consumer preference (Desmond and Kenny 1998; Wang and Xiong 1999; Murphy et al. 2004).

In this study, we developed a frankfurter-type sausage with 91.25% of MSTM with a high acceptation. In another study, sausages prepared with minced fish (MF) from Nile tilapia *O. niloticus* filleting waste had a maximum recommended MF of only 60% inclusion to maintain good sensory quality (Oliveira Filho et al. 2010).

## Conclusions

Developing low-fat or reduced-fat meat products is especially challenging for comminuted meat products because reducing fat often results in changes in palatability of the meat products. However, the frankfurter-type sausages from mechanically separated tilapia surimi produced and characterized here present promising characteristics for commercial applications. Moreover, it was not possible to obtain frankfurter-type sausages from TV surimi due to technological problems observed during cooking.
